# The UK ME/CFS Biobank: A Disease-Specific Biobank for Advancing Clinical Research Into Myalgic Encephalomyelitis/Chronic Fatigue Syndrome

**DOI:** 10.3389/fneur.2018.01026

**Published:** 2018-12-04

**Authors:** Eliana M. Lacerda, Kathleen Mudie, Caroline C. Kingdon, Jack D. Butterworth, Shennae O'Boyle, Luis Nacul

**Affiliations:** Department of Clinical Research, Faculty of Infectious and Tropical Diseases, London School of Hygiene and Tropical Medicine, London, United Kingdom

**Keywords:** ME/CFS, research infrastructure, biobank, partnership, patient engagement PE

## Abstract

Myalgic encephalomyelitis/chronic fatigue syndrome (ME/CFS) is a disabling disease characterized by unexplained incapacitating fatigue, accompanied by variable multi-systemic symptoms. ME/CFS causes a significant personal and public health burden, and urgently requires the coordination of research efforts to investigate its etiology and pathophysiology and to develop and validate sensitive and specific biomarkers to confirm diagnosis. This narrative paper describes how people with ME/CFS, together with a multidisciplinary team of researchers, have established the UK ME/CFS Biobank (UKMEB), a unique research infrastructure specifically designed to expedite biomedical research into ME/CFS. We describe the journey that led to its conceptualization and operation, and how the resource has served as a model disease-specific biobank, aggregating human biospecimens alongside comprehensive health information on participants. The UKMEB currently has data and samples from 600 donors including people with ME/CFS and a comparison group with multiple sclerosis and healthy controls. A longitudinal sub-cohort has been established of participants having follow-up assessments at multiple time-points. As an open resource for quality and ethical research into ME/CFS, biological samples and data have not only been analyzed within our research team but have also been shared with researchers across Europe, America and the Middle East. We continue to encourage researchers from academic and commercial sectors to access the UKMEB. Major steps have been taken and challenges remain; these include sustainability and expansion, and harmonization of processes to facilitate integration with other bioresources and databanks internationally.

## Introduction

This paper describes a journey, beginning with extensive conversations between medical researchers, people with myalgic encephalomyelitis/chronic fatigue syndrome (ME/CFS), their carers, ME charities, and a multidisciplinary team of professional experts, and continuing with the establishment and operation of the UK ME/CFS Biobank (UKMEB) (1).

The UKMEB is, to our knowledge, the only biorepository in the United Kingdom, and one of few worldwide, dedicated to the study of ME/CFS. Such disease-specific biobanks hold high quality anonymised samples enriched by comprehensive datasets with information about the donors, all appropriately and securely stored until required for use in research. We delineate the progress our team have achieved and the remaining challenges that need to be addressed in order for the UKMEB to be able to realize its full potential.

Using a combination of qualitative methods (1, 2), we carried out extensive consultations with people with ME/CFS (PWME), a multidisciplinary group of experts in tissue banking, ethics and law, and clinicians and researchers with expertise in ME/CFS. The resultant unanimous view was that a disease-specific biobank would be a highly desirable way to enhance biomedical research in ME/CFS. In the safety of the participatory environment, PWME were able to express their justifiable concern that, in the absence of biomarkers, their illness is frequently dismissed as trivial or psychosocial (3–5), even though many are more debilitated by their disease than people with other chronic and severe diseases such as cancer and rheumatoid arthritis (6, 7). PWME told us that they would be willing to donate tissues, including blood, oral fluid and urine for research, as part of a process centered on the needs and priorities of those with ME/CFS (1). They believed that an ME/CFS Biobank would be feasible and cost-effective, and that its implementation would strengthen and further ME/CFS research.

## Features of the UKMEB Protocol

The input from both PWME and the multidisciplinary group led to the development of a robust protocol, incorporating recommendations, which included:

- Comprehensive patient phenotyping and depth of any information provided by biobank donors;
- The use of rigorous standards for data and sample collection, processing, and storage;
- The inclusion of patients who are “*severely affected, including those that are bed-ridden*” (quote from a person with ME/CFS during focus-groups discussions); and
- The inclusion of control or comparison group(s).

Participants of the UKMEB were recruited through the National Health Services (NHS) general practices (GPs) and specialist services with support from the clinical and research networks of the National Institute of Health Research (NIHR). Those with ME/CFS required a previous medical diagnosis of ME/CFS and those with MS a previous diagnosis given by a NHS consultant. Healthy controls were also recruited through GP practices, other participants' contacts and higher education institutes.

The UKMEB received ethical approval from the London School of Hygiene & Tropical Medicine (LSHTM) Ethics Committee (ref. 6123), the National Research Ethics Service (NRES) London-Bloomsbury Research Ethics Committee (REC; ref. 11/LO/1760, IRAS ID: 77765), and the NHS Research Governance and Developments Offices (R&D), which oversee the recruitment of research participants from government health services. LSHTM and UCL-RFH Biobank hold Human Tissue Act licenses—HTA-12066 and HTA-11016, respectively.

Possible barriers to participation in a potential biobank resource were also discussed, and were mostly related to concerns with the misuse of the resource:

“*if you're trying to get as much people as you can, they are afraid of what you're going to do, whether the government would get a hold of it, whether the insurance companies could use it, or whether benefit agencies would use it”* (quote from a person with ME/CFS during focus-groups discussions).

It was agreed that such misuse could be avoided by the implementation of robust ethical standards, which is reflected in the UKMEB mission statement that reads—“*The UKMEB is to conduct high quality, ethical investigations into ME/CFS and to create an open resource to enable translational research for the clinical and biomedical understanding of the illness while fostering cooperation and collaboration between researchers and thereby enhancing the opportunity for breakthrough discoveries*.”

Other key aspects of the final protocol informed by PWME together with experts include:

### Control Groups

In addition to ME/CFS cases of different severities, we have recruited donors who serve as healthy controls; these individuals are grouped matched by age and sex and have no history of fatigue or fatigue-causing diseases, including cancer, hepatitis B or C, major depression or psychiatric illness, obesity, and diabetes. We also recruited people with Multiple Sclerosis (MS)—who often experience chronic fatigue as a major symptom—for a disease comparison group.

### Clinical Phenotyping

Detailed questioning of potential participants with ME/CFS enables their disease to be classified according to different case definitions. To be accepted as a participant with ME/CFS, potential donors must meet either the Canadian Consensus Criteria (8) or CDC-1994 criteria (9); many fulfill both. The assessment process for compliance with study criteria includes baseline questionnaires about symptoms, a clinical assessment performed by a clinical member of the research team, and urinalysis screening and baseline blood tests, which are used to exclude alternative diagnoses.

In some association studies bias can be minimized by using samples from participants who meet at least both sets of criteria (10).

Extensive data collection together with the results of molecular analyses facilitates disease stratification, which aims to identify subgroups of patients with distinct mechanisms of disease (or other features), which may require quite different treatment approaches. Initial sub-grouping can be based on readily available variables obtained from patient questionnaires, examples of which include age, sex, type of disease onset (e.g., sudden or gradual; post-infection or not), co-morbidities, clinical severity, and disease phase and duration. The “Participant Phenotyping Questionnaire” completed by all UKMEB participants has been used to characterize individuals according to the presence and severity of seven groups of symptoms (or symptom clusters), which are largely based on the Canadian Consensus Criteria (8). Table 1 compares study groups according to some of these variables, including general indicators of disease severity and the severity of symptoms related to each of the clusters described. For the latter, the scores are obtained from the severity of individual symptoms, each expressed as a value from 0 to 3; scores from each symptom within the cluster are added together, resulting in the cluster score, which is adjusted on a scale from 0 to 100, where “0” represents no symptoms and “100” symptoms experienced with maximum severity. For example, the severity of post-exertion malaise symptoms is highest in the severely affected cases of ME/CFS (median = 80), also high in those with mild/moderate disease (median = 67), and modest in people with MS (median = 27).

**Table 1:** Characteristics of cases (both mild/moderately affected and severely affected) and controls (healthy controls and MS diseased controls) within UKMEB, with cases of ME/CFS defined using a combination of three diagnostic criteria: CDC-1994, CCC, and IOM.

	**Characteristic**		**Cases**			**Controls**	
			**CDC94+CCC+IOM** ***N* = 232(38)**	**Mild/ moderately affected^**†**^** ***N* = 177(76)**	**Severely Affected‡** **N = 55(24)**	**HC** ***N* = 153(25)**	**MS *N* = 90** **(15)**
	Age, in years	Median(IQR)	48 (38,56)	48 (40,57)	50 (37,55)	47 (35,56)	55 (48,60)
	Sex, female	N(%)	155 (76)	114 (76)	41 (76)	84 (62)	59 (78)
	Disease duration (years)	Median(IQR)	12 (6, 18)	10 (5, 17)	16 (9, 22)	-	12 (8, 20)
Disease Severity^*^	Fatigue severity scale	Median(IQR)	6.7 (6.3,7.0)	6.7 (6.2,7.0)	6.7 (6.3,6.9)	2.0 (1.6,2.8)	5.7 (4.1,6.4)
	Fatigue analog scale	Median(IQR)	7.3 (6.2,8.2)	6.9 (6.1,7.8)	7.6 (6.6,8.3)	1.1 (0.2,2.1)	6.2 (3.6,7.2)
	Pain analog scale	Median(IQR)	5.8 (3.2,7.2)	5.8 (3.1,7.1)	5.9 (3.2,7.4)	0.5 (0.0,1.2)	2.8 (0.7,6.2)
	PCS	Median(IQR)	26 (20,33)	28 (24,36)	20 (16, 22)	58 (56,60)	38 (29,48)
	MCS	Median(IQR)	41 (33,49)	40 (32,46)	46 (38,51)	55 (49,58)	48 (39,55)
Severity score for clusters of symptoms^**^	Post-exertional Malaise	Median(IQR)	67 (53,67)	67 (53,67)	80 (73,80)	0.0 (0.0,0.0)	27 (13,53)
	Pain	Median(IQR)	47 (27,67)	53 (27,67)	47 (27,80)	3.5 (0.0,10)	20 (7,40)
	Neurological/cognitive symptoms	Median(IQR)	50 (33,67)	43 (31,60)	62 (50,83)	0.0 (0.0,5.0)	36 (21,52)
	Autonomic	Median(IQR)	40 (23,57)	37 (22,50)	52 (40,77)	0.0 (0.0,3.0)	19 (10,32)
	Neuroendocrine	Median(IQR)	47 (33,67)	47 (27,60)	60 (47,80)	0.0 (0.0,0.0)	33 (20,53)
	Sleep dysfunction	Median(IQR)	83 (67,100)	67 (67,100)	100 (67,100)	0.0 (0.0,33)	50 (25,67)
	Immune	Median(IQR)	33 (22,50)	33 (22,50)	33 (22,56)	0.0 (0.0,6.0)	6.0 (0.0,11)
Disease onset	Suddenly	N(%)	91 (46)	60 (42)	31 (58)	-	30 (39)
	Over time	N(%)	80 (41)	63 (44)	17 (32)	-	31 (41)
	Not sure	N(%)	25 (13)	20 (14)	5 (9)	-	15 (20)

*CDC94: Centres for Disease Control-1994; CCC: Canadian Consensus Criteria; IOM: Institute of Medicine; HC: healthy controls; MS: Multiple Sclerosis; PCS: Summary Physical Component Score from SF-36; MCS: Summary Mental Component Score from SF-36v2^TM^*.

* *For Fatigue Severity and Analog Scales and Pain Analog Scales; values vary from 0 to 10, where 10 indicates maximum severity. Normalized Physical and Mental Component summaries are presented; higher values represent better health status/quality of life*.

** *Severity of symptoms within the cluster; values range from 0 (no symptom) to 100 (most severe symptoms)*.

† *Mild/moderately affected defined as participants who are ambulatory*.

*‡Severely affected defined as participants who are house- or bed-bound*.

### Inclusion of the Severely Affected

The systematic inclusion of participants with very severe ME/CFS for research purposes is, we believe, unique to the UK ME/CFS Biobank. This patient group usually has poor access to services and has often been excluded from research studies, not only because they are home- or bed-bound, but also because PWME often disengage from statutory medical services when they encounter skepticism or when the treatment offered is of limited value. Reaching them involves complex logistic and economic considerations.

“*Arranging to see these extremely ill participants presents its own challenges, including the timing of appointments and the length of time that it may take to clinically assess participants, whose every move can take an enormous effort and for whom the process can require days of preparation and weeks of recovery time. Any external stimuli including touch, light and sound can exacerbate symptoms, so strategies must be undertaken to reduce the impact on participants. Certain clinical assessment procedures may not be feasible and blood samples are sometimes taken with the light from a torch in a darkened room. Nonetheless, the materials generated by these severely affected participants could provide crucial insight into the pathology of the disease, as they may present with exaggerated biochemical and/or immunological changes.”* (Quote from CK (co-author) on the task of the Research Nurse)

### Longitudinal Data and Samples

Through the systematic longitudinal collection of clinical data and blood samples, it is possible to investigate associations between clinical characteristics and changes in disease severity over time, as well as in a range of molecular markers, e.g., immune and genetic expression phenotypes.

We employ several validated measures of disease severity (11), while acknowledging the need for further development of ME/CFS-specific outcome measures. For example, patient-based assessments of disease impact or severity, such as the SF-36v2^TM^, have been used in a variety of clinical settings and are monitored in clinical research to add to our understanding of disease severity, treatment outcomes, and therapeutic response (12). Changes between baseline and follow-up assessment-points can be compared with differences in biomarkers to help characterize clinical phenotypes. By using the suggested minimally important difference (MID) in SF-36v2^TM^ normalized scores, e.g., of ±4.7 points for the Physical Component Summary (PCS) and ±5.8 points for the Mental Component Summary (MCS), it is possible to ascertain improvement or worsening of scores with 95% confidence (12).

**Figure 1:**
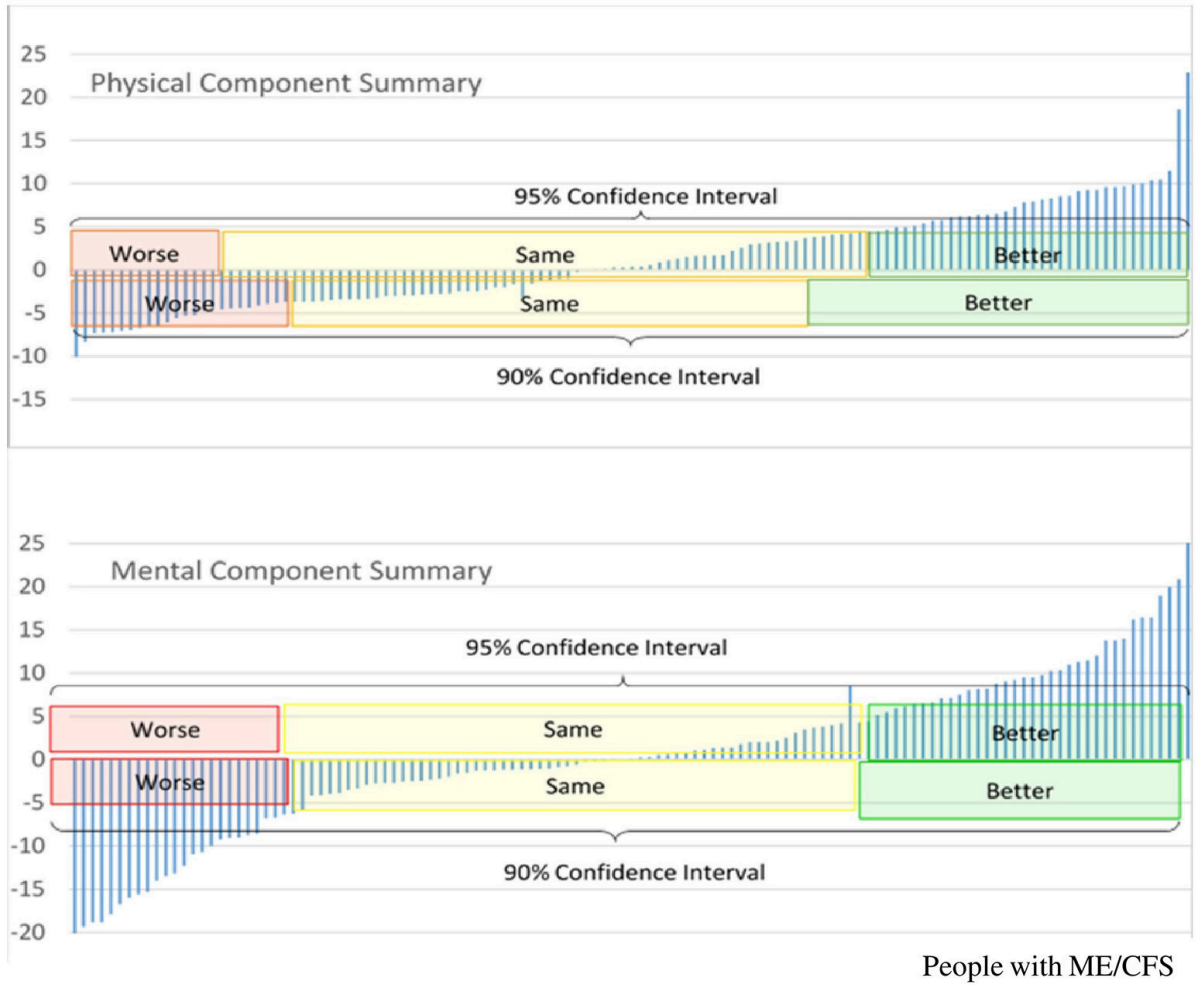
Longitudinal changes in SF-36v2^TM^ Component Summary scores, in people with ME/CFS who participate in the UK ME/CFS Biobank, between baseline and first follow-up^*^. ^*^First follow up occurred between 6 and 12 months from baseline, Y-axis represents difference in scores between first follow-up assessment and baseline assessment; positive values indicate scores are higher at follow-up assessment, and therefore represent improvement. PCS, Normalized Physical Component Summary scores from SF-36v2^TM^; MCS, Normalized Mental Component Summary score from SF-36v2^TM^.

Figure 1 shows that <50% of the PWME demonstrated significant changes in these indicators from baseline to subsequent assessment. If there are persistent trends toward improvement or deterioration in repeated assessments, these may reflect disease progress or pathophysiological changes that may differ from those related to a fluctuation of symptoms.

## UKMEB Potential: Current Status and Enhancing Research

### Resource Sharing

Between 2013 and 2017, biological samples alongside questionnaire and clinical data were collected from 600 participants (including 350 PWME), forming an extensive dataset. A second round of data collection took place 6–12 months after recruitment, with 140 PWME and 130 controls followed-up after baseline. From 2018 to 2020, a further 650 participants are planned to be seen at least once and a subset of 110 PWME to be seen on at least four occasions (and up to six), creating robust data that enables powerful longitudinal analyses.

The UKMEB currently holds over 35,000 aliquots of blood derivatives. Blood taken from each participant is processed to produce seven different types appropriate to the expected end use of the samples and suitable for a wide range of assays. After fractioning, an average of 46 aliquots is stored following each participant-contact as follows: serum (*n* = 10), plasma (processed from sodium heparin vacutainers *n* = 7, and from EDTA vacutainers *n* = 3), peripheral blood mononuclear cells (PBMCs) (processed from sodium heparin vacutainers *n* = 17, and from EDTA vacutainers *n* = 3), whole blood (*n* = 4), and RNA (*n* = 1–2). Additionally, for each participant contact, the UKMEB stores red blood cells/granulocyte pellet (*n* = 1), and PAXGENE tubes (*n* = 1–2).

Some of these are available to researchers at the LSHTM, the home of the UKMEB team, contributing to their ongoing projects in immunology, genomics, transcriptomics, virology, and clinical research. The rest of the samples are stored for the use of researchers from the academic and commercial sectors in biomedical research, following an established protocol for the release of samples, subject to ethical review and an approved, peer-reviewed application https://cureme.lshtm.ac.uk/researchers/accessing-the-biobank/.

Biobanks facilitate the sharing of biological samples and data in a cost- and time-effective way over many years (13). The cost savings to researchers vary depending on multiple factors, such as the type and size of the study, but have been estimated to provide a 90% saving (14). These are in addition to significant time savings in selection, recruitment, and data and sample acquisition. This centralization of data and samples creates economies of scale, enabling and accelerating research.

In May 2016, the UKMEB opened to external researchers, who are able to apply for access to samples and data. Academic, non-commercial, and commercial researchers have since been eligible to apply to use the Biobank, when the proposed study has a sound scientific rationale and all ethical permissions are in place. Priority of research applications is given to studies testing or generating new hypotheses on the pathophysiology of ME/CFS, improving diagnosis and phenotyping, or in basic science (e.g., pharmacological *in vitro* studies potentially leading to clinical trials on therapeutic approaches).

The procedures for this access to data and samples, approved by the UKMEB Biobank Steering Committee, a multidisciplinary body comprising PWME, carers, researchers, and clinicians, include: (i) a review of outline proposals by Steering Committee members, (ii) a peer review of full proposals, (iii) ethical approval by the UCL-RFH ethics committee (BERC), and (iv) Data and/or Material Transfer Agreements (DTA and/or MTA). All proposals must also receive ethical approval from their local ethics committee.

Since the UK ME/CFS Biobank opened its doors, it has received applications from institutions in the UK and other European countries, North and South America, and the Middle East - encompassing diverse research topics including immunology, metabolomics, genetics, transcriptomics, and microbiology.

### Sustainability

The UKMEB has relied on support from charities and has benefited from research grants, which have helped with recruitment, sample acquisition, data entry, and storage. This funding has supported core infrastructure, but only for the time period in which projects were taking place. To survive and thrive in the long term, plans were made for the continuing storage of samples and data, and particularly for the release of samples to external researchers. This necessitated the creation of a UKMEB Business Plan, which evolved with input from the Steering Committee.

A fee structure was calculated and agreed upon - fees are requested from biobank users on a cost-reimbursement basis, so that sample stores can be replenished in the future and, where possible, additional recruitment or follow-up can be facilitated.

The long-term sustainability of the UKMEB relies upon multiple income streams, minimizing the risk of being exposed overly to any one source of funding. In financial year August 2015—July 2016, charity funding formed around 90% of gross revenue, but in financial year August 2017–July 2018, that had reduced to around 60%, with donations and cost recovery charged forming the remaining of 40% of total gross. The key elements of future UKMEB income are (i) fees for using the samples and data, (ii) philanthropic donations, (iii) crowdfunding and regular giving, and (iv) research grant support, which have to coexist to ensure long-term sustainability (14, 15). The opening of other income streams in the past financial year has shown that it is possible to move to a multi-source revenue system in biobanking, once appropriate start-up capital has been invested to enable fundraising and awareness-raising efforts.

### Engagement With the Community

Social media, websites and web fora play a fundamental role in the lives of many with ME/CFS, facilitating social connection with others in the community as well as with researchers and charities, particularly when the physical demands of face-to-face interaction are not feasible. For PWME, online platforms can be one of the few places where their voices are heard and can be an invaluable resource in encouraging ongoing partnership between the ME/CFS community and researchers.

Engagement with these communities remains a key pillar of the UKMEB's strategy. We endeavor to remain transparent in our research objectives and stay engaged with PWME online, so that the communities we serve are actively involved in how we progress. In addition, we strive to be accountable to our donors by sharing whatever news and findings we can via social media and via our website.

This renewed focus on public engagement (since August 2017) has coincided with an increase in our donation income, as well as an increase in applications received from researchers wanting to use our resource. While any concrete causal relationship is difficult to prove, we feel that an active and open public engagement strategy supports several of the income streams delineated above, and helps contribute to the UKMEB's sustainability efforts, while also building trust between the research team and the community.

## Final Remarks—Where This Journey Is Heading?

Biobanks are recognized as key to biomedical research; and their numbers have been increasing globally, as human bio-specimens, combined with health information on their donors, provide a critical resource for biomedical research (16). Disease-specific biobanks, in particular, are useful for addressing conditions such as ME/CFS, where there remain important unanswered questions around causes, diagnosis, pathophysiology and treatment (17, 18). We believe that the UKMEB can be used as a model for others contemplating developing bio-resources in the field of ME/CFS; or indeed for other specific diseases, one that incorporates participatory approaches, partnership, time and cost-effectiveness, and sustainability into the design and implementation.

The integration of the UKMEB with other ME/CFS-specific biobanks could involve the sharing of protocols or at least an agreement to collect some common data and samples, and will be essential for accelerating much-needed ME/CFS research. Such research will likely include the investigation of potential biomarkers, transcriptomics, metabolomics, genomic and genetic studies; biobanks may also be accessed by those seeking to improve diagnosis and treatment, and undertake validation studies.

The poor recognition of and stigma that surrounds the disease are still present, and the wider needs of PWME in relation to healthcare, social, occupational and education support remain largely unmet (3). We have previously described the perceived needs of PWME, and some aspects of the care they receive (19). In this article, we focused on how the UKMEB evolved as we sought, in partnership, to help address some of these needs and to advance research into the disease. We described a journey that evolved from conversations with people with ME to an established resource facilitating biomedical research into ME/CFS locally and internationally. The journey has only just begun.

The enthusiasm from the ME/CFS community and from participants, including those with MS and healthy controls, has contributed to the success of a project developed while keeping the needs of patients and the research community in mind. With follow-up rates presently over 90% and an increasing number of external researchers using data and samples, the UKMEB is successfully delivering. However, the real benefits will only be felt when the results of research are effectively translated into better health for PWME.

There is no doubt that ME/CFS research can further be accelerated through the integration of bio-resources and the facilitation of consistent data collection globally. We are actively discussing and engaging with other bio-resources globally. The Common Data Elements project developed by the National Institute of Neurological Disorders and Stroke (20), is one example of an initiative aimed at data harmonization and integration in ME/CFS research. Another is the European Network on ME/CFS (EUROMENE), which combines resources, technologies, and expertise from over 20 European countries in a multidisciplinary approach to optimize knowledge production in the field. The harmonization of ME/CFS related data and bio-resources across the continent is one of the objectives of the network (21).

Improved research is only one of many challenges that needs addressing in the field, and we hope that our experiences presented here represent some contribution to this effort. It is only with substantial increases in research and research-infrastructure funding, and significant improvement in services for those with ME/CFS, that the individual journeys of PWME will be improved.

## Author Contributions

EL, LN, KM, and CK conceptualized the article, KM, LN, and EL conducted the clinical and epidemiological analyses. All authors contributed to drafting and to revising the manuscript critically for important intellectual content. All authors approved the final version of the manuscript to be published.

### Conflict of Interest Statement

The authors declare that the research was conducted in the absence of any commercial or financial relationships that could be construed as a potential conflict of interest.
